# The heat sensitive factor (HSF) of *Yersinia ruckeri* is produced by an alkyl sulphatase involved in sodium dodecyl sulphate (SDS) degradation but not in virulence

**DOI:** 10.1186/s12866-014-0221-7

**Published:** 2014-09-30

**Authors:** Roberto Navais, Jessica Méndez, Desirée Cascales, Pilar Reimundo, José A Guijarro

**Affiliations:** Área de Microbiología, Departamento de Biología Funcional, Facultad de Medicina, IUBA, Universidad de Oviedo, 33006 Oviedo, Asturias Spain

**Keywords:** *Yersinia ruckeri*, HSF factor, Alkyl sulphatase, Virulence

## Abstract

**Background:**

The heat sensitive factor (HSF) of the fish pathogen *Yersinia ruckeri* was previously identified as an unusual band on SDS-PAGE. According to this, *Y. ruckeri* strains were classified in HSF^+^ and HSF^*−*^ in terms of the presence/absence of the factor. Experiments carried out by injection challenge with HSF^*+*^ strains caused high mortalities in rainbow trout. In contrast, HSF^*−*^ strains did not cause mortality. In conclusion, HSF appeared to be a relevant virulence factor in *Y. ruckeri*.

**Results:**

We report here the identification and study of the gene coding for the enzyme involved in the production of HSF. Culture medium containing SDS and Coomassie brilliant blue dye was used to screen a mini-*Tn5 Km2* mutant library of *Y. ruckeri* 150. Blue colonies lacking a surrounding creamy deposit, a phenotype described in former studies as HSF^**−**^, were identified. DNA sequence analysis of a selected mutant revealed that this had a transposon interruption in a chromosome-located gene which codes for a heat sensitive alkyl sulphatase of 78.7 kDa (YraS; *Yersinia ruckeri *alkyl *s*ulphatase) which is able to degrade SDS to 1-dodecanol. As it was expected, the introduction of the *yraS* gene into an HSF^**−**^ strain turned this into HSF^**+**^. Surprisingly, although the protein allows *Y. ruckeri* to degrade SDS, the bacterium could not use this compound as the sole carbon source. Moreover, the yraS mutant showed a similar level of SDS resistance to the parental strain. It was the interruption of the *acrA* gene which made *Y. ruckeri* sensitive to this compound. LD_50_ experiments showed a similar virulence of the yraS mutant and parental strain.

**Conclusions:**

The HSF of *Y. ruckeri* is the product of the alkyl sulphatase YraS, able to degrade SDS to 1-dodecanol. This degradation is not linked to the utilization of SDS as a carbon source and surprisingly, the enzyme is not involved in bacterial virulence or in the high SDS resistance displayed by the bacterium. This role is played by the AcrAB-TolC system.

**Electronic supplementary material:**

The online version of this article (doi:10.1186/s12866-014-0221-7) contains supplementary material, which is available to authorized users.

## Background

*Yersinia ruckeri* is a Gram-negative bacterium alternating between planktonic and host interaction states. It is the causative agent of enteric redmouth disease (ERM) affecting mainly salmonids in fish farms, which leads to important economic losses in aquaculture worldwide. Four serological groups [1] and two biotypes [2] of *Y. ruckeri* are currently proposed. Amongst them, serotype 1 is the most virulent, being commonly isolated from outbreaks in fish farms. Whereas the mechanisms involved in the virulence of human pathogenic *Yersinia* species have been studied in depth, only a few pathogenic mechanisms of *Y. ruckeri* have been described [3–8]. In this context, of special interest is the work of Furones et al. [9], who found an association between the virulence of *Y. ruckeri* serotype I strains and the presence of a heat-sensitive factor (HSF), identified as a sodium dodecyl sulphate polyacrylamide gel electrophoresis (SDS-PAGE) band of approximately 120 kDa from cell extracts. The strains were classified in HSF^**+**^ and HSF^**−**^ in terms of the presence/absence of the factor [9]. Experiments carried out by injection challenge with HSF^**+**^ strains caused high mortalities in rainbow trout. In contrast, HSF^**−**^ strains did not cause mortality. These authors concluded that HSF appeared to be a relevant virulence factor in *Y. ruckeri*. In order to be able to distinguish HSF^+^ and HSF^−^ strains routinely, Furones et al. [10] developed a differential culture medium containing SDS and the Coomassie brilliant blue dye. In this medium, the colonies of the HSF^+^ strains appeared as white, due to a creamy deposit around the colony, whereas those of HSF^**−**^ strains did not form this deposit and became deeply blue.

Given the relationship between the presence of the HSF and the virulence of *Y. ruckeri*, we decided it would be interesting to investigate the gene coding for this factor. The HSF was found to be produced by an alkyl sulphatase, a protein which is not related to the virulence of the bacterium, but with potential usefulness for removing surfactant from the environment.

## Results

### Creamy white *Y. ruckeri* colony phenotype (HSF^+^) growing in the presence of SDS is caused by an alkyl sulphatase (YraS)

**Figure 1:**
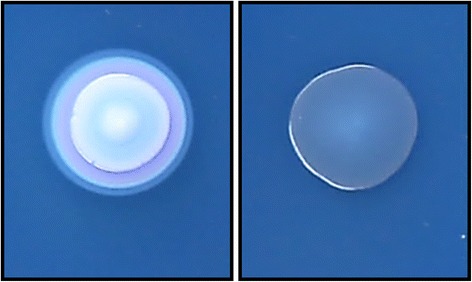
**Colony morphology of** ***Y*** **.** ***ruckeri*** **strains grown on TSA-SDS containing Coomassie brilliant blue dye showing the HSF** ^**+**^ **(** ***Y*** **.** ***ruckeri*** **150 parental strain, left) and HSF** ^**−**^ **(mutant obtained after the screening of a** ***Y*** **.** ***ruckeri*** **150** **Tn5 Km2** **library, right) phenotypes.** Aliquots of 5 μl from early stationary phase cultures of the parental and mutant strains were spotted onto TSA-SDS Coomassie brilliant blue medium. After 48 h of incubation at 28°C colonies were photographed. Creamy white colony (HSF^+^) contrasts with the blue colony (HSF^**−**^) phenotype.

**Figure 2:**
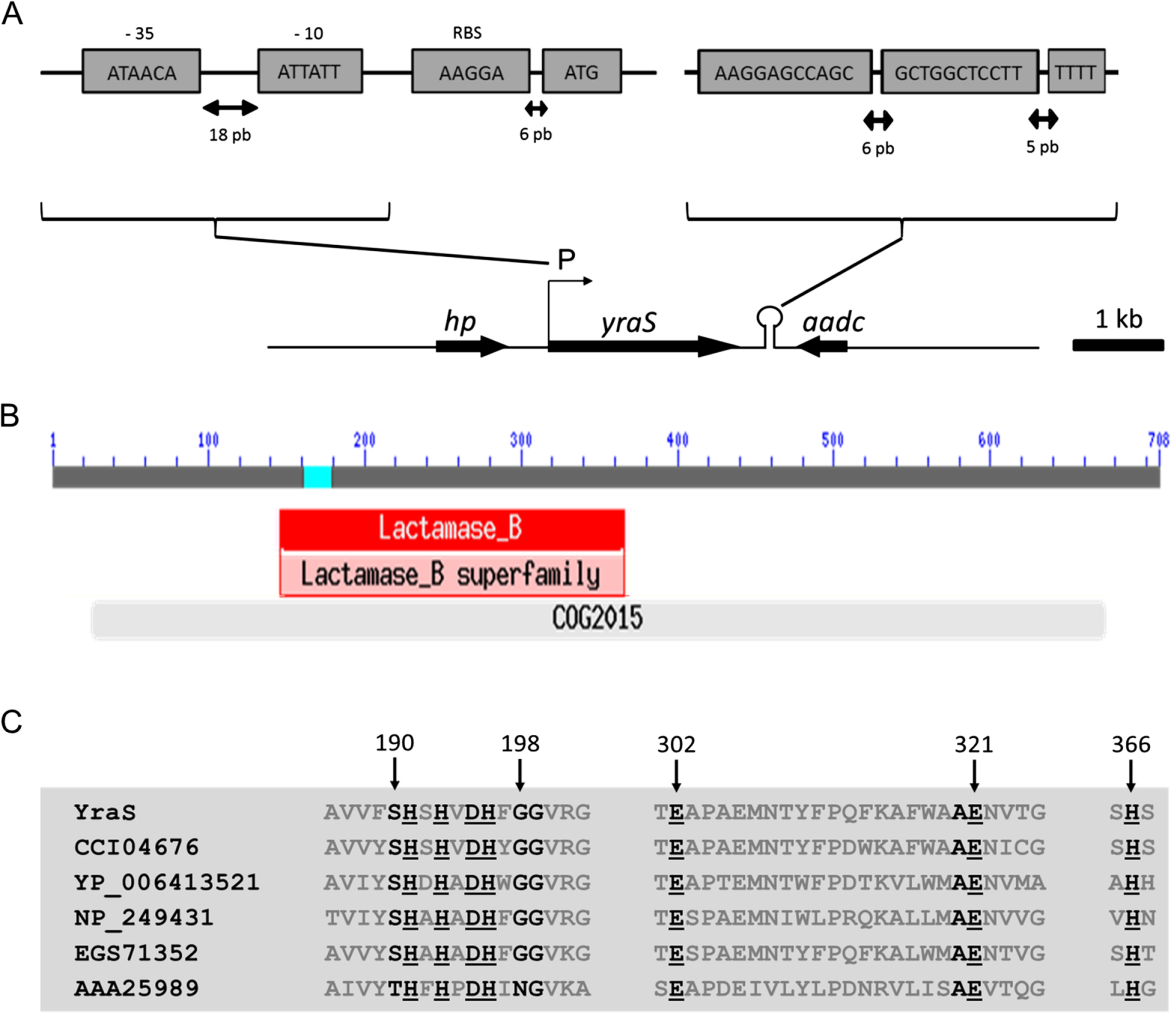
**Gene organization of the** ***yraS*** **locus and protein domains of the gene product. (A)** The position of each gene and the direction of transcription are shown by arrows. The position and sequences of the *yraS* putative promoter (P), RBS, rho-independent transcriptional terminator (hairpin loop) are indicated. The flanking genes *hp* (hypothetical protein) and *aadc* (aromatic amino acid decarboxylase) are also shown. **(B)** Structural organization and domains of the YraS protein showing the COG2015 (light grey line) and the β-lactamase domain (red cassette). A blue mark inside the β-lactamase domain shows the position of a compositionally biased region not used in domain database search. **(C)** Sequences alignment of the Zn^2+^ binding domain from YraS homologue proteins. Amino acid sequences correspond to position 186 to 367. The amino acids involved in Zn^2+^ binding are underlined. Yras, *Y. ruckeri*; CCI04676, *Microcystis aeruginosa*; YP_006413521, *Thiocystis violascens*; NP_249431, *P. aeruginosa*; EGS71352, *Vibrio cholerae*; and AAA25989, *Pseudomonas* sp. ATCC19151.

A mini-*Tn5 Km2 Y. ruckeri* transposon library was screened for colonies lacking the creamy white deposit around the colonies on TSA medium containing SDS and Coomassie brilliant blue (Figure 1). Southern blot and sequence analysis of the interrupted genes in several blue coloured colonies showed that all of them presented a unique chromosomal transposon insertion in a 2,127 bp open reading frame. Putative −10 (ATTATT) and −35 (ATAACA) promoter sequences, and ribosome–binding site (AAGGA) were identified upstream of the ORF, and a stem-loop palindromic sequence corresponding to a rho-independent terminator was located at the 3′end of the gene (Figure 2).

The product of the interrupted gene, a protein of 708 amino acids, shows a high degree of identity with other proteins defined as alkyl sulphatases or SDS hydrolases from different bacteria such as *Microcystis aeruginosa* (71%) (CCI04676), *Thiocystis violascens* (63%) (YP_006413521), *Vibrio cholerae* (52%) (EGS71352) and *Pseudomonas aeruginosa* (51%) [11].

*In silico* analysis indicated that the protein carries an N-terminal signal peptide of 24 amino acid residues and contains two domains: the cl00446, characteristic of the metalo-β-lactamase family, and the COG2015, typical of alkyl sulphatases (Figure 2). Both domains are present in the SdsA and SdsA1 alkyl sulphatases from *Pseudomonas* sp. ATCC19151 (AAA25989) [12] and *P. aeruginosa* PAO1 (NP_249431) [11], respectively. Additionally, the protein harbours, at its N-terminal sequence, a Zn^2+^-binding motif (T**H**x**H**x**DH**xGG-102-**E**-18-**AE**-44-**H**) characteristic of metallo-β-lactamase-related enzymes, also present in the SdsA and SdsA1 alkyl sulphatases [11,12] (Figure 2).

The *in silico* results were in concordance with data obtained from the analysis by gas chromatography–mass spectrometry of the creamy-white compound produced by cultures of the *Y. ruckeri* parental strain grown in NB with 0.25% SDS. The compound was identified as 1-dodecanol (Additional file 1: Figure S1), the molecule resulting from the hydrolysis of SDS by an alkyl sulphatase [13].

The phylogenetic tree, based on the YraS protein of different microorganisms, showed that *Y. ruckeri* does not share any evolutionary origin with the bacteria harbouring an YraS-homologous protein (Additional file 2: Figure S2). However, all the bacteria that are closer to *Y. ruckeri* in the phylogenetic tree, with the exception of *Sinorhizobium meliloti*, are also aquatic. One example is *Photobacterium profundum*, a psychrophilic marine bacterium belonging to the Vibrionaceae family. Proteins homologous to YraS were also found in other Enterobacteriaceae species such as *Klebsiella oxytoca*, *Klebsiella variicola* and *Salmonella enterica*. However, the proteins of these bacteria are very distant in the phylogenetic tree from the YraS protein of *Y. ruckeri* (Additional file 2: Figure S2).

### YraS is the HSF factor

**Figure 3:**
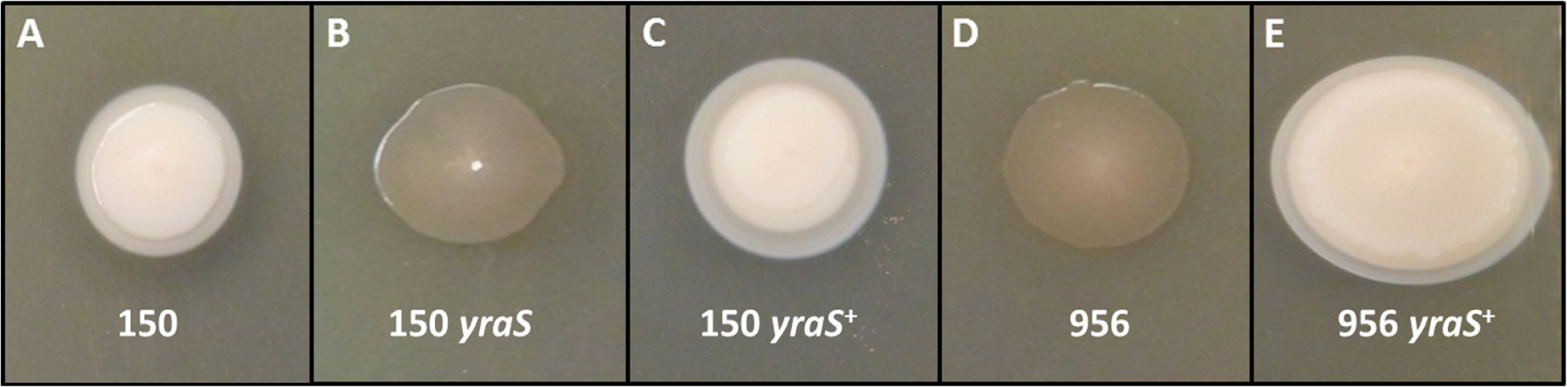
**Colony morphology of different** ***Y*** **.** ***ruckeri*** **strains grown on TSA-SDS showing the HSF** ^**+**^ **and HSF** ^**−**^ **phenotypes.** Strains were point inoculated and after 120 h at 28°C colonies were photographed. **A**. *Y. ruckeri* 150 parental strain. The HSF^−^ phenotype of yraS mutant (HSF^−1^) **(B)**, and 956 strains **(D)** changes to HSF^+^ phenotype (**C** and **E**, respectively), when they are complemented by the *yraS* gene.

In order to go further into the relationship between the *yraS* gene and the HSF^**+**^ phenotype, defined by a creamy deposit around the colony on SDS containing media (Figure 3A), one of the obtained mutants showing an HSF^**−**^ colony phenotype (HSF^−1^; Figure 3B) was complemented with the *yraS* gene. As can be observed in Figure 3C, a complete reversion of the HSF^**−**^ to HSF^**+**^ colony phenotype was achieved. Furthermore, when the *yraS* gene was introduced in *Y. ruckeri* 956, a serotype II strain showing an HSF^**−**^ phenotype, the colony phenotype changed to HSF^**+**^ (Figure 3D,E). Both, yraS mutant and 956 strains, when complemented with the *yraS* gene, originated a larger creamy deposit than that of the parental strain, probably owing to gene dosage effect (Figure 3C,E). In conclusion, the *yraS* gene is responsible for the HSF^+^ phenotype of *Y. ruckeri* colonies on SDS-containing media.

**Figure 4:**
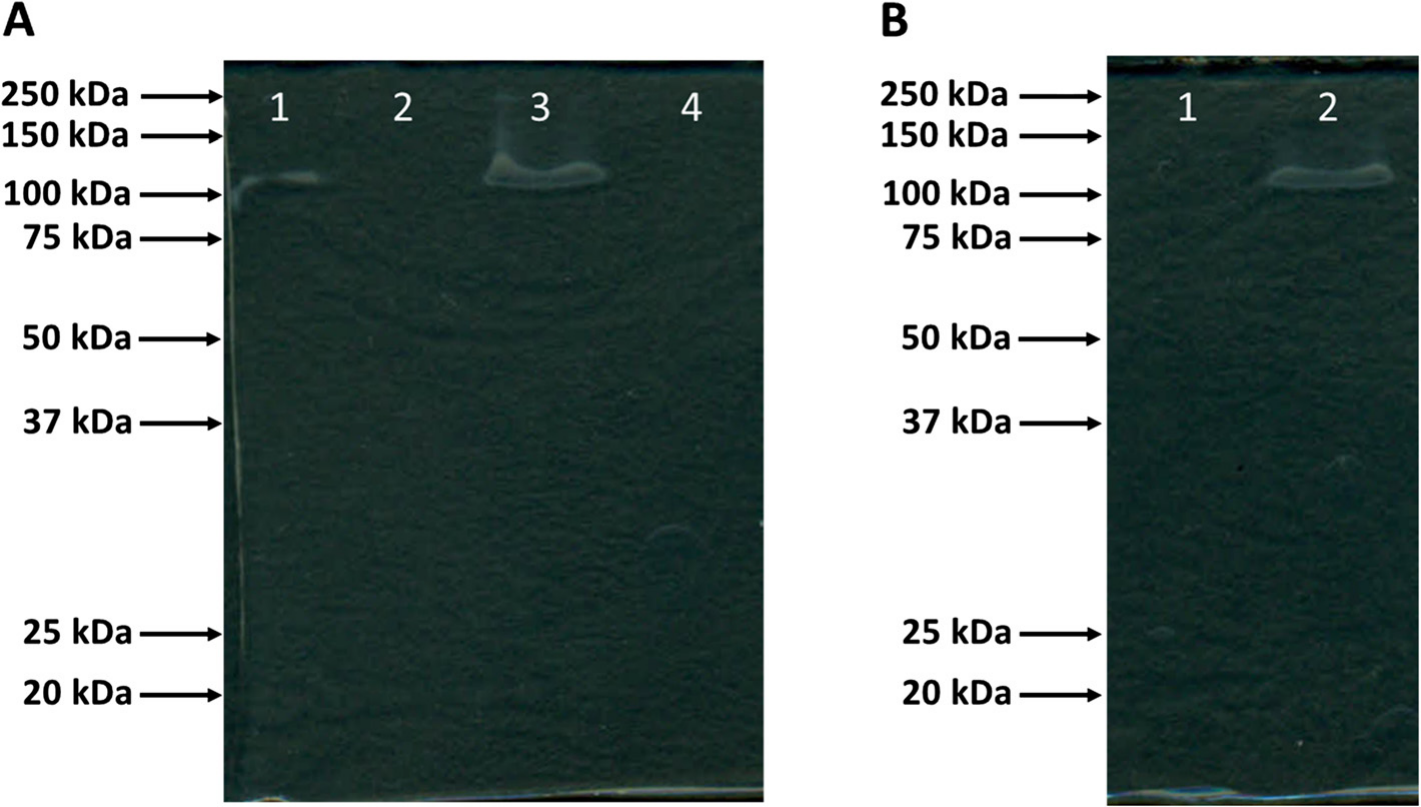
**Zymograms showing SDS hydrolysis after SDS-PAGE of crude extract of different** ***Y*** **.** ***ruckeri*** **strains.** SDS-PAGE of bacterial cell extracts was performed at 15 mA in a cool room for 16 h. Then, gels were incubated at 20°C for 4 h and kept 1 additional hour at 4°C for SDS precipitation. Bands of SDS hydrolysis activity appear as clear zones against an opaque gel. **(A)** Cell extract from: lane 1, *Y. ruckeri* (parental strain); lane 2, yraS^−^; lane 3, yraS^+^; lane 4, parental strain heated at 100°C for 10 min. **(B)** Cell extract from: lane 1, *Y. ruckeri* 956; lane 2, 956yraS^+^. Molecular mass markers (in kDa) are indicated on the left side of each gel. Photographs were taken on a dark background to contrast the bands. There was a match between the appearance in the SDS-PAGE of the 120 kDa SDS hydrolysis bands and the presence of the *yraS* gene in the strains.

To confirm that the YraS protein was linked to the HSF factor, defined by Furones et al. [9] as a 120 kDa band in SDS-PAGE gels, cell extracts from the different *Y. ruckeri* parental, yraS mutant, 956 and complemented strains were obtained and separated by SDS-PAGE under the conditions previously described [9]. Figure 4A, lane 1, shows the presence in the *Y. ruckeri* parental strain of a band, resulting from the degradation of the SDS present in the gel, with an apparent molecular mass of approximately 120 kDa. This band disappeared in the yraS mutant (Figure 4A, lane 2) and returned when the mutant was complemented with the *yraS* gene (Figure 4A, lane 3). This band was also absent in the HSF^**−**^*Y. ruckeri* 965 strain (Figure 4B, lane 1), and again appeared when this strain was *yraS* complemented (Figure 4B, lane 2). The presence in the gel of this 120 kDa band was temperature-dependent. Indeed, when the cell extracts of the HSF^**+**^ strains were heated to 100°C for 10 min, before SDS-PAGE separation, the 120 kDa band was absent (Figure 4A, lane 4). Moreover, when the gel was stained with Sudan black dye the 120 kDa band became stained (Additional file 3: Figure S3). Both types of behaviour were defined previously for the HSF factor [9].

### HSF^+^/HSF^−^ phenotype correlates with the presence/absence of the *yraS* gene in *Y. ruckeri* strains

**Figure 5:**
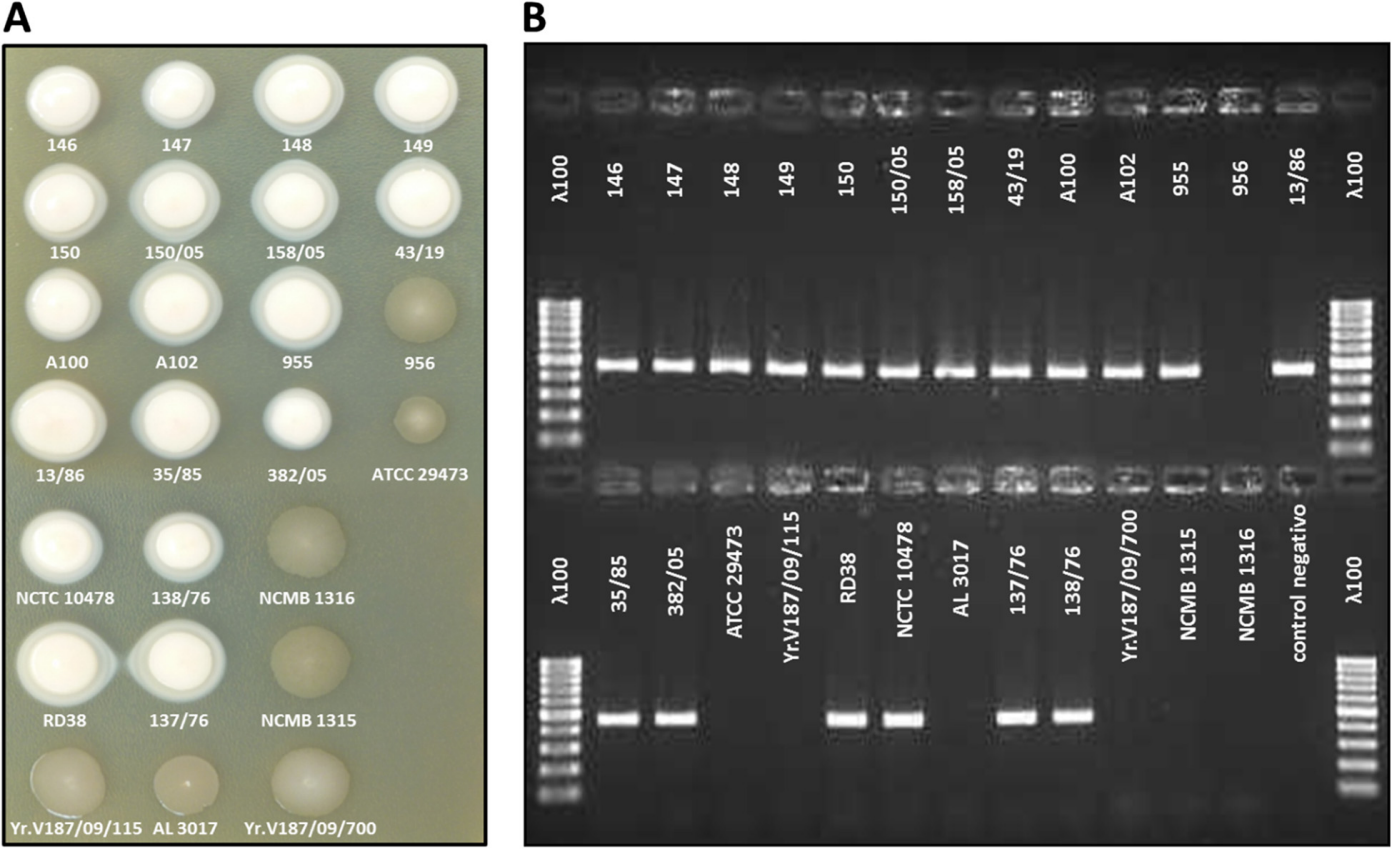
**Relationship between HSF** ^**+/−**^ **colony phenotype and presence/absence of the** ***yraS*** **gene in different** ***Y*** **.** ***ruckeri*** **isolates. (A)** Aliquots of 5 μl of early stationary phase cultures of isolates of *Y. ruckeri* from different hosts and geographic origins were spotted onto TSA-SDS medium. After 48 h of incubation at 28°C, colonies were photographed. **(B)**. Aliquots from the same cultures were used for an *yraS* gene fragment amplification using PCR reactions according the protocol described in Experimental Procedures. PCR-generated amplicons were separated in agarose gel and photographed. Lane ʎ100 shows DNA molecular mass markers from 100 to 1000 bp. All the isolates showing HSF^+^ phenotype (creamy white colonies) were positive for the PCR reaction, whereas the isolates with HSF^−^ phenotype (transparent colonies) did not present the amplicon.

A set of *Y. ruckeri* strains, including some used in the study of Furones et al. [9,10], were analysed by PCR for the presence of the *yraS* gene and also tested for colony morphology on SDS-containing medium. In all the cases, there was a match between colony morphology and the presence of the *yraS* gene: all the HSF^+^ strains harboured the *yraS* gene and presented a white creamy deposit surrounding the colonies, whereas in HSF^**−**^ strains the *yraS* gene was absent and colonies lacked the white creamy deposit (Figure 5).

### The *yraS* gene is not involved in virulence and it has no nutritional role

The presence in *Y. ruckeri* of the HSF factor, now defined as the product of the YraS enzyme, was previously associated with virulence [9,10]. In order to define the role of this factor in virulence, LD_50_ experiments were carried out in groups of 10 rainbow trout fish (weight 10–15 g) intraperitoneally injected with 100 μl of parental and yraS mutant strains dilutions from 10^2^ to 10^8^ CFU/ml. Fish death occurred along the 7 days period and cumulative mortality curves were similar for both strains in each dilution (Additional file 4: Figure S4). At the end of this period, LD_50_ values obtained were 1.0 × 10^2^ CFU (with lower and upper 95% confidence limits of 1.3 × 10 and 4.0 × 10^2^ CFU), and 1.1 × 10^2^ CFU (with lower and upper 95% confidence limits of 9.0 and 4.8 × 10^2^ CFU), respectively. These results showed that the YraS protein is not a virulence factor.

Constitutive expression of the *yraS* gene seems to occur, since the YraS protein is present in both NB and M9C media. A first approach to the role of the YraS protein was inferred from experiments in which glucose was added to the M9C medium containing 1% (w/v) SDS. After 96 h of incubation at 28°C, no degradation at all of SDS by the parental strain occurred when glucose was present in the culture medium. In the same way, the parental strain showed an HSF^**−**^ phenotype when glucose was present in M9C medium containing SDS (Additional file 5: Figure S5). This result could be a consequence of a catabolic repression mechanism involved in the regulation of the *yraS* gene. Therefore, it would be possible that *Y. ruckeri* uses SDS as an additional carbon source for bacterial growth. However, when the different *Y. ruckeri* strains were incubated in M9 solid and liquid media containing different amounts of SDS as sole carbon source no growth at all was observed. Definitely, *Y. ruckeri* SDS degradation by YraS is not linked to the utilization of this detergent as a sole carbon source. Moreover, the presence in the culture media of different amounts of sulphate did not produce any visible alteration in SDS degradation, suggesting that sulphate is not involved in the regulation of the *yraS* gene.

### The protein YraS is not involved in SDS-resistance; instead, this property lies within the AcrAB-Tolc system

**Figure 6:**
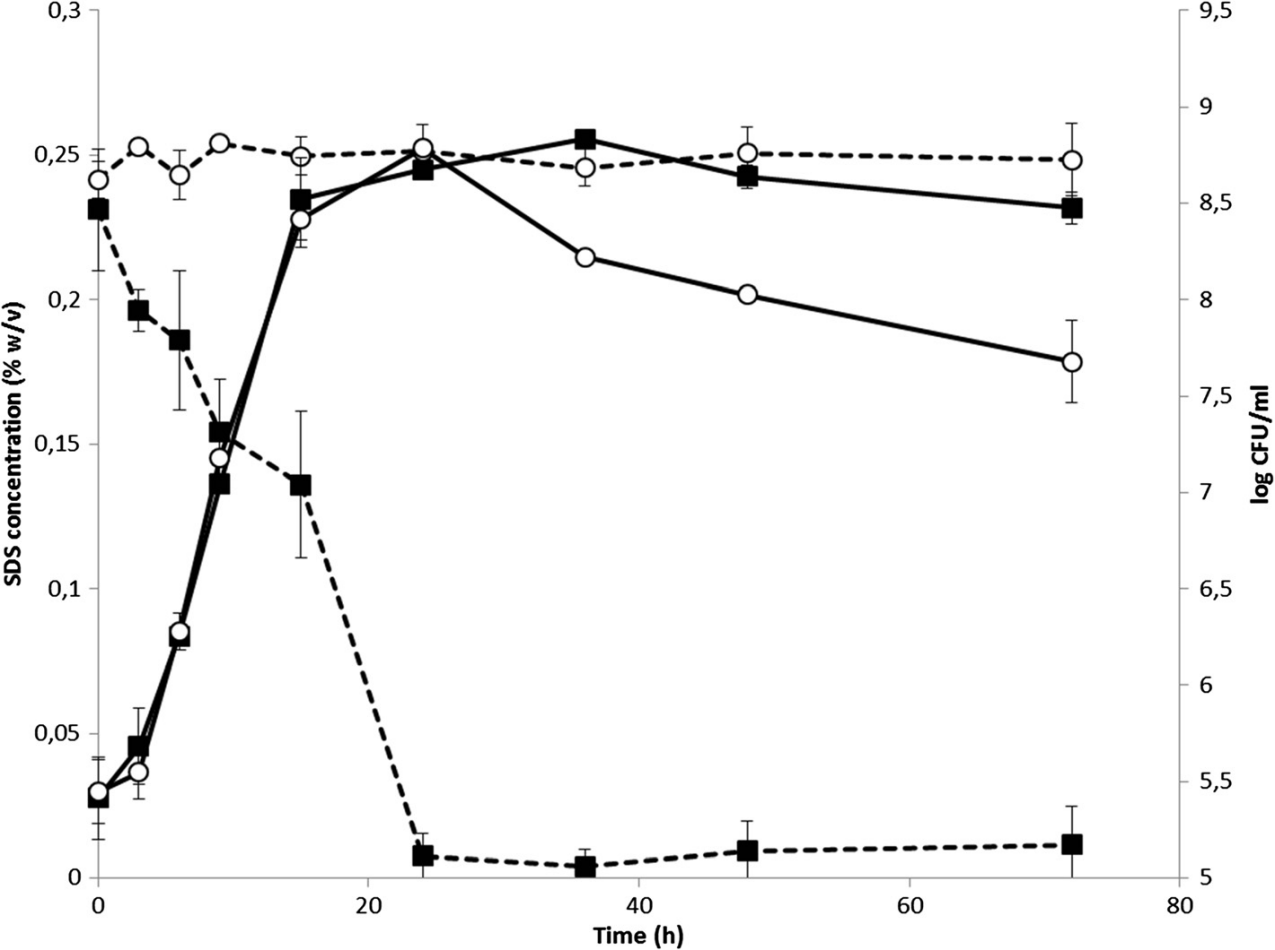
**Growth curves and SDS degradation by** ***Y*** **.** ***ruckeri*** **parental and yraS mutant strains.** Cultures were incubated in NB containing 0.25% (w/v) SDS at 28°C and 250 rpm and, at different times, samples were withdrawn and growth determined by plate counting. Simultaneously, the presence of SDS in the culture supernatant was quantified by the Stains-all method [14]. Continuous line and dotted line represents the growth and SDS degradation of parental (■) and yraS^−^ (○) strains. Data represent the mean ± standard deviation of three independent experiments. Growth was similar for the two strains, but SDS was completely degraded by the parental strain after 24 h of incubation, whereas no degradation at all occurred by the yraS mutant strain.

Growth of the yraS mutant strain in NB medium containing 0.25% SDS at 28°C was similar to that of the parental strain, although a greater decrease in cell viability occurred in the mutant strain during the stationary phase of growth (Figure 6). SDS was totally degraded by the parental strain after 24 h of incubation at 28°C, but no degradation at all occurred during the growth of the yraS mutant (Figure 6). Percentages of 0.5% and 1% SDS were not totally degraded by the parental strain, probably because nutrient depletion occurred before all the detergent was hydrolysed.

To examine the role of the YraS protein in the physiology of the bacterium, the MIC of SDS for different *Y. ruckeri* strains was determined. The results indicated that an identical level of resistance to SDS (MIC=3.2% w/v) was found amongst *Y. ruckeri* parental, yraS mutant and complemented *yraS*^**+**^ strains. In addition, no major resistance to SDS was found when the *Y. ruckeri* 956 strain was complemented with the *yraS* gene, the MIC for both strains was 0.8% (w/v). These results indicated that the alkyl sulphatase is not a relevant SDS-resistance mechanism in the bacterium. Similarly, both parental and mutant strains presented the same MIC for Triton X-100, Tween 80 and bile salts (data not shown).

In order to elucidate the system responsible for the high SDS resistance of the bacterium, a mini-Tn5 Km2 mutant library of *Y. ruckeri* was screened to select a mutant unable to grow in the presence of 0.5% SDS. The gene whose interruption was responsible for that phenotype codes for a protein homologous to AcrA, one of the components of the AcrAB-TolC system. This is involved in pumping out of the cell a wide variety of compounds including detergents and antibiotics in bacteria such as *Escherichia coli* and *Salmonella enterica* [15–17]. Phenotypic analysis of this mutant showed that it was sensitive to SDS, having a MIC of 0.00625% w/v, instead of the 3.2% w/v of the parental strain. Additionally, the *acrA* mutant was significantly more sensitive than the parental strain to Triton X-100, bile salts and the antimicrobials tetracycline, oxitetracycline, ciprofloxacin, chloramphenicol and oxolinic acid (data not shown).

## Discussion

Virulence in *Y. ruckeri* was previously correlated with the presence of the HSF^**+**^ colony phenotype [9]. In this work, this HSF^**+**^ phenotype was linked to the product of the *yraS* gene which corresponds to an alkyl sulphatase. This enzyme is involved in the degradation of alkyl sulphate esters, such as SDS, generating as a first product water insoluble 1-dodecanol. This compound, which, in *Y. ruckeri* is accumulated in the medium, forms a white creamy deposit around colonies and leads to a band of 120 kDa in SDS-PAGE gels. Both phenotypes were previously observed by Furones et al., [9,10]. According to *in silico* analysis, the YraS enzyme falls into a new class of alkyl sulphatases characterized by the presence of a metallo-β-lactamase domain and found mostly in gram-negative bacteria [11]. This kind of enzymes cleaves alkyl sulphates such as SDS into the corresponding alcohol.

The YraS activity was identified in SDS-PAGE as a diffuse SDS-hydrolytic band of 120 kDa, approximately. This result is coincident with the molecular mass of HSF described by Furones et al. [9]. According to this, and taking into account that the molecular mass of the YraS, deduced from the amino acid sequence, was 78.7 kDa, the active form of the enzyme should correspond to a protein dimer structure. This also agrees with the dimeric structure of the active form of the SdsA1 from *P. aeruginosa*, which provides resistance to high concentrations of SDS [11]. In the same way, a lipid core structure for HSF was suggested, since the 120 kDa band was stained with Sudan black, a dye used for lipid detection [9]. Our results showed that the 120 kDa band was also stained with this dye. Therefore, the lipidic core structure of HSF suggested by Furones et al. [9] was correct, since it is likely that the compound stained with Sudan black is the fatty alcohol 1-dodecanol, the product of the alkyl sulphatase action on SDS. Nevertheless, we must link the HSF to the alkyl sulphatase rather than to the product of SDS degradation by the enzyme. This fact is consistent with the absence of the band when the cell extract was heat-inactivated before electrophoresis. Comparative analysis of parental and yraS mutant *Y. ruckeri* strains, complementation studies, as well as PCR detection of the *yraS* gene in different strains of *Y. ruckeri*, confirm that YraS is responsible for the production of the HSF factor. These results, together with the presence of a single 120 kDa band in the cell extracts of the parental *Y. ruckeri* strain, strongly suggested that, under these experimental conditions, there were no additional alkyl sulphatases involved in the degradation of SDS, and the YraS protein was the only one responsible for this activity. This differs from different reports that indicated that bacteria able to degrade alkyl sulphate esters possess multiple sulphatase enzymes [11,18-22].

Our results indicate that there was no relation between the presence of HSF factor and virulence in *Y. ruckeri*. Indeed, results of the rainbow trout infection experiments carried out with the parental and yraS mutant strains, showed that both strains behaved similarly as virulent bacteria. These results are not incompatible with the one obtained by Furones et al., [9,10] because although YraS is not a virulence factor its presence could be linked to the existence in the bacterium of specific virulence factors. In this way, YraS could be a hallmark of virulence even though it is not involved in pathogenesis. This could explain why HSF^+^ strains are always virulent [9,10].

The YraS protein was produced by the bacterium when growing in media lacking SDS and therefore, it seems that the gene is constitutively expressed. However, YraS activity was absent when the bacterium was grown in the presence of glucose, suggesting a regulation of the *yraS* gene by catabolic repression. Nevertheless, the absence of bacterial growth in the presence of SDS as a sole carbon source as well as the accumulation of 1-docecanol in the culture media, indicate that *Y. ruckeri*, in contrast to *P. aeruginosa* [11], is unable to use this detergent as sole carbon source. 1-dodecanol, resulting from SDS degradation by the YraS protein, is likely to enter the bacterium where it would be oxidized to 1-dodecanoic acid by the action of the appropriate alcohol dehydrogenase and aldehyde dehydrogenase to be assimilated as a carbon source. Therefore, it seems that the absence of one or more of these systems could be a problem which prevents further metabolic assimilation of 1-dodecanol by *Y. ruckeri*.

No alteration in detergent hydrolysis by *Y. ruckeri* was observed when inorganic sulphate was added to the media. Our results suggest that in *Y. ruckeri* this enzyme is not involved in the sulphur cycle and is not a system for scavenging sulphate from the environment. Whereas bacterial aryl sulphatases have been involved in sulphate scavenging [23], alkyl sulphatases seem to have additional roles [11,24,25]. In conclusion, and according to the results, it seems that the *yraS* gene is not related to carbon or sulphur utilization.

There are many reports of bacteria able to degrade different amounts of SDS. For example, a *P. putida* SP3 strain was able to completely degrade 0.1% SDS in 16 h [26]. However, most of the SDS-degrading bacteria isolated presented lower rates, needing 4 to 10 days of incubation to completely degrade percentages of 0.05 to 0.2% of SDS [27,28]. Interestingly, *Y. ruckeri* degrades 0.25% SDS in NB within 24 h of incubation at 28°C, and can grow in the presence of up to 1.6% of this detergent. Introduction of the *yraS* gene in *Y. ruckeri* strains lacking the capacity to degrade SDS enables these strains to do so. However, *Y. ruckeri* SDS resistance is not *yraS*-dependent since the growth curve of the yraS mutant strain in the presence of 0.25% SDS was similar to that of the parental strain. To our knowledge, it is here established for the first time that a gene involved in SDS degradation is not associated with the resistance of the bacterium to this detergent. Usually it is assumed that the presence of alkyl sulphatases in a particular bacterium is related to its resistance to toxic alkyl sulphate molecules. However, in fact, resistance of *Y. ruckeri* to SDS lies in the AcrAB-TolC system, a pump involved in the excretion of multiple compounds such as antimicrobials and detergents in different bacteria [15–17], as was indicated by the analysis of an SDS-sensitive *Y. ruckeri acrA* mutant. Therefore, in this particular case, and probably in other bacteria bearing this kind of sulphatase-coding gene, it may be that the *yraS* gene is only present in naturally SDS resistant *Y. ruckeri* strains. These characteristics, high SDS resistance and degradation capacity, make this bacterium potentially useful as a tool for removing anionic surfactants from different sources.

It is interesting to speculate about the presence of this enzyme in a bacterium alternating between planktonic and host interaction states. The results indicate that the protein does not have a significant role in the infectious process or in nutrition or detergent resistance. However, the constitutive expression in nutritionally rich and poor media suggests that the protein provides an advantage to the bacterium in its natural environment. The high resistance to SDS depending on the AcrAB-TolC system, together with the high rate of degradation of this compound by the alkyl sulphatase YraS, reinforces the potential usefulness of bacteria possessing high expression systems of both mechanisms in the fight against surfactant pollution in bioremediation.

## Conclusions

We have showed in this work the nature of the previously defined HSF factor of *Y. ruckeri* as the product of SDS degradation by the YraS alkyl sulphatase. Although in former works it was established a correlation between the possession of HSF and the virulence of this microorganism, our results indicate that the factor is not necessary for bacterial pathogenicity. YraS was able to degrade SDS but it does not contribute, at least in a relevant way, to the high SDS resistance observed in this bacterium. This function corresponds to the AcrAB-TolC system.

## Methods

### Bacterial strains and culture conditions

The bacterial strains and plasmids used in this study are listed in Tables 1 and 2, respectively. *E. coli* strains were routinely grown in 2xTY broth and agar, and *Y. ruckeri* strains in nutrient broth (NB) and nutrient agar (NA). Screening of *Y. ruckeri* strains for the HSF^+^/HSF^−^ colony phenotype was performed on TSA medium supplemented with 1% SDS (TSA-SDS). Coomassie brillant blue dye was added to molten TSA-SDS medium to provide final concentrations of 100 μg/ml, as described by Furones et al., [10]. Experiments on SDS as a nutrient were performed using the minimal medium described by Romalde et al. [29] without glucose (M9) and M9C (M9 containing 2 g/l casamino acids) broth and agar media supplemented with different percentages of SDS. When it was required glucose was added to M9C to a concentration of 0.5% (w/v). For motility experiments, a semisolid tryptone agar medium (5 g/l NaCl, 10 g/l tryptone, 0.6% w/v agar) was used. Liquid cultures were incubated at 37°C for *E. coli* and 18°C and 28°C for *Y. ruckeri* in orbital shakers at 250 rpm. Growth was monitored by determining the OD_600_. When SDS was present in the liquid culture growth was determined by serial dilutions and plate counts. When required, the following compounds were added to the media: 100 μg/ml ampicillin, 0.1 μg/ml cephotaxime, 50 μg/ml kanamycin or streptomycin.

**Table 1:** **Bacterial strains used in this study**

**Strain**	**Serotype**	**HSF**	**Other characteristics**	**Source of reference**
***Y*** **.** ***ruckeri***				
150	I	+	Isolated during outbreaks of ERM disease in Denmark	J.L. Larsen, University of Frederiksberg (Denmark)
150 *acrA*	I	+	*acrA*::mini-Tn5 Km2 Kmr	This study
150 *yraS*	I	-	*yraS*::mini Tn5 Km2 Kmr	This study
150 *yraS*+	I	+	150 *yraS* harboring pGBM5-*yraS*	This study
146,147,148,149	I	+	Isolated during outbreaks of ERM disease in Denmark	J.L. Larsen, University of Frederiksberg (Denmark)
955	I	+	Trout-isolated strain	CECT (Spanish Type Culture Collection)
956	II	-	Trout-isolated strain	CECT (Spanish Type Culture Collection)
43/19	I	-	Trout-isolated strain	CECT (Spanish Type Culture Collection)
956 *yraS*+	II	+	956 harboring pGBM5-yraS	This study
35/85*	I	+	Isolated from *Salmo gairdneri* in Denmark	C.J. Rodgers (University of Tarragona, Spain)
13/86*	I	+	Isolated from *Salmo gairdneri* in England	C.J. Rodgers (University of Tarragona, Spain)
A100, A102,	I	+	Trout-isolated strain	I. Márquez (SERIDA, Spain)
150/05,158/05,382/05	I	+	Trout-isolated strain	Proaqua Nutrition S.A.
137/76*,138/76*	I	+	Trout-isolated strain	C.J. Rodgers (University of Tarragona, Spain)
NCTC 10478*	I	+	Isolated from *S. gairdneri* in USA	C.J. Rodgers (University of Tarragona, Spain)
NCMB 1315*	NT	-	Isolated *from S. gairdneri* in USA	C.J. Rodgers (University of Tarragona, Spain)
NCMB 1316*	I	-	Isolated from *S. gairdneri* in USA	C.J. Rodgers (University of Tarragona, Spain)
Yr.V187/09/115*	I	-	Isolated from *S. salar* in Norway	C.J. Rodgers (University of Tarragona, Spain)
Yr.V187/09/700*	II	-	Trout-isolated strain	C.J. Rodgers (University of Tarragona, Spain)
ATCC 29473*	I	-	Isolated from *S. gairdneri* in USA	C.J. Rodgers (University of Tarragona, Spain)
RD38*	I	+	Trout-isolated strain	R.L. Davies, University of Stirling (Scotland)
AL 3017	NT	-	Trout-isolated strain	M.D. Furones, IRTA (Tarragona)
***E*** **.** ***coli***				
DH5αλ*pir*	NT	NT	F’/endA1 hsdR17 (rk-mk+) supE44 thi-1 recA1 gyrA (NalR) λ (*pir*)	[30]
S17-1λ*pir*	NT	NT	λ (*pir*) hsdR pro thi, RP4-2 Tc::Mu Km::Tn7	[31]

*Strains used in the work of Furones *et al*., [9,10].

**Table 2:** **Plasmids used in this study**

**Plasmid**	**Characteristics**	**Source or reference**
pGBM5	Spc^r^/Sm^r^, lac promoter	[32]
pGBM5-*yraS*	pGBM5 harboring *yraS* gene	This study
pUC19	Ap^r^, cloning vector	Pharmacia
pUT mini-Tn5 Km2	Ap^r^, oriR6K, mobRP4, *tnp*, mini-Tn Km2 (Km^r^)	[33]

### Mutant selection, DNA sequencing and mutant complementation

A mini-Tn5 Km2 transposon based *Y. ruckeri* 150 mutants library was generated by using the pUT mini-Tn5 Km2 plasmid. This plasmid was transferred by conjugation from *E. coli* S17 ʎ1*pir* to *Y. ruckeri* 150 strain [34]. Transconjugants were first selected on NA plates supplemented with kanamycin and cephotaxime by incubation for two days at 28°C. Approximately 10000 transposon mutants were replicated onto TSA-SDS medium containing Coomassie brillant blue dye and incubated for five days. Colonies showing an HSF^**−**^ phenotype characterized by a deep blue colour were selected. For testing that selected transconjugants presented a sole transposon insertion, selected colonies were analysed by Southern blotting using a DIG DNA detection kit (Roche) following the protocol described by the manufacturer. Total DNA from transconjugants and the parental strain was isolated (GenElute Mammalian Genomic DNA Purification Kit-Sigma) and digested with *Eco*RI and *Xba*I restriction enzymes. Then, DNA fragments were subjected to 0.75% w/v agarose gel electrophoresis at 100 V for 2 h, transferred to a nylon membrane (Amersham Biosciences) and fixed with UV irradiation. The labelled region of the kanamycin gene from the Tn5 transposon was used as a probe to perform hybridization. For genome location of the mini-Tn5 transposon mutated gene, plasmid was obtained by the Kado and Liu [35] method, and total DNA was extracted from the *Y. ruckeri* strains 150 and 956 using GenElute Mammalian Genomic DNA Purification Kit-Sigma. DNA extracted from both procedures was separated by agarose gel electrophoresis and after transfer to nylon membranes, submitted to hybridization using as a probe a labelled 455 bp PCR-generated internal fragment of the *yraS* gene. PCR was performed using the following primers: yraS-a (5′-ACCGAAGCGCCAGCAGA-3′) and yraS-b (5′-AGTGTCGCTGGATTACC-3′).

The *acrA* mutant was obtained after replica plating of a mini-Tn5 transposon library on TSA medium and TSA containing 0.5% w/v SDS. Colonies able to grow on TSA but unable to do so in the presence of SDS were selected. Unique transposon insertion in the genome of the bacterium was assessed by Southern blot analysis as described above using as a probe the labelled region of the kanamycin gene from the Tn5 transposon.

To obtain the complete sequence of *yraS* (the gene responsible for the HSF^**+**^ phenotype), genomic DNA from the *Y. ruckeri* yraS mutant strain was digested with *Eco*RI and *Pst*I restriction enzymes to obtain the DNA regions adjacent to the 5′ and 3′ ends, respectively, from the kanamycin gene of the mini-Tn5 Km2 transposon. The restriction fragments were ligated into the pUC19 plasmid previously digested with the corresponding enzymes and dephosphorylated, and the mixture was used to transform cells of *E. coli* S17-1λ*pir* by electroporation. Transformants were selected on 2 × TY agar medium containing kanamycin and ampicillin. Plasmid DNA was obtained and sequencing was carried out using primers from the mini-Tn5 Km2 transposon sequence (Tn5-sec:5′-AAACGCGTATTCAGGCT-3′, and Tn5-sec2:5′-GCGTATTCAGGCTGACC-3′). Based on the obtained sequences new primers were designed to complete the *yraS* gene sequence. DNA sequencing was performed by the dideoxy chain termination method with the BigDye Terminator version 3.1 (Applied Biosystems) according to the manufacturer's instructions in an ABI Prism 3730 DNA sequencer.

A similar procedure was performed for the sequencing of the *acrA* gene. The DNA from the selected mutant was digested with *Kpn*I and *Xba*I restriction enzymes for the sequencing of the DNA region located at 5′ and 3′position with respect to the kanamycin gene. After DNA digestion, the fragments were ligated into the pUC19 plasmid and the mixture was used to transform cells of *E. coli* S17-1λ*pir* by electroporation. From this point the same procedure described in the previous paragraph was followed.

To complement the *Y. ruckeri* yraS mutant and *Y. ruckeri* 956 strains, the *yraS* gene was amplified from the parental strain by PCR using Biotools DNA polymerase and primers yras-S (5′-CCTG*GTCGAC*GGTTGGTATTGTCTGGT-3′) and yras-E (5′-GGCG*GAATTC*AGTCAGTGAGATAACGA-3′). The *Sal*I and *Eco*RI restriction sites (in italics) were introduced into the sequences of yras-S and yras-E, respectively, to clone the PCR product digested with *Sal*I and *Eco*RI into pGBM5 [32] previously digested with the same enzymes. The resulting plasmid was designated pGBM5–*yraS* (Table 2). Transfer of pGBM5-*yraS* to 150*yraS* and 956 strains was carried out by electroporation and colonies were selected on 2xTY agar medium containing streptomycin. The plasmid was recovered from the transformants, digested by *Sal*I and *Eco*RI and analysed by agarose gel electrophoresis to confirm the presence of the insert.

### SDS-PAGE analysis of the HSF presence in *Y. ruckeri* strains

*Y. ruckeri* cultures from the different strains were grown in NB until the early stationary phase of growth. Bacterial cultures were centrifuged at 13,000 rpm for 10 min and cells re-suspended in 62.5 mM Tris–HCl (pH 6.8). Cells were broken by ultrasound treatment in an ice bath, samples were then centrifuged at 13,000 rpm for 30 min at 4°C and the supernatants were used for SDS-PAGE electrophoresis. A 1:3 volume of 2x Laemmli sample buffer was added to the samples and then they were subjected to SDS-PAGE in 12% gels in a cool room overnight at 15 mA. Gels were then incubated at 20°C for four hours and finally screened for white opaque bands, a product of the degradation of the SDS contained in the gel, over a dark background. When needed, samples were treated at 100°C for 10 min before SDS-PAGE. Sudan black staining was performed after gel electrophoresis by immersion of the gel in a Sudan Black solution (0.5% w/v) in ethanol. The gels were examined for blue bands after destaining.

### PCR detection of the *yraS* gene in different *Y. ruckeri* strains

The presence of the *yraS* gene in a variety of *Y. ruckeri* strains from different origins and geographic areas was examined by PCR using the following primers: yraS-a (5′-ACCGAAGCGCCAGCAGA-3′) and yraS-b (5′-AGTGTCGCTGGATTACC-3′). The amplification reaction was performed in a Perkin-Elmer 9700 GeneAmp thermocycler with an initial denaturation cycle at 94°C for 5 min, followed by 25 cycles of amplification (denaturation at 94°C for 30 s, annealing at 52°C for 1 min, and extension at 72°C for 1 min), and a final 7-min elongation step at 72°C. The presence of a 455 bp amplicon was confirmed by agarose gel electrophoresis.

### Gas chromatography–mass spectrometry of SDS derivative product and SDS quantification

Stationary-phase cultures of *Y. ruckeri* parental and yraS mutant strains were used to inoculate (1:100) 250 ml Erlenmeyer flasks containing 20 ml of NB supplemented with SDS (0.25% w/v). A similar flask containing NB was used as control. After 24 hours of incubation at 28°C and 250 rpm, cultures were filtered through disc filters with a pore size of 0.45 μm (Pall Life Sciences). The filtrates were dried in a speed vac and re-suspended in 1 ml of chloroform. Samples were then subjected to a gas chromatography–mass spectrometry system consisting of an Agilent model 6890 N-5975B (Santa Clara, California, USA) equipped with a capillary column Agilent 19091 J-433 HP-5 (30 m × 0.25 mm, 0.25 μm film thickness, Agilent Technologies, California, USA). A 1-min splitless injection of 1 μl of a 1:400 dilution of each sample was used. Linear velocity of the carrier gas (helium): 36 cm/s (1 ml/min). Oven program: 40°C, 20°C/min to 250°C; 10°C/min to 300°C (hold for 5 min). Temperatures of injector, source, interface and quadrupole were 270°C, 230°C, 280°C y 150°C, respectively. 70 eV was used for ionization. Mass spectra were recorded scanning the 20–550 m/z range.

For SDS quantification, at different times during the growth curve samples were withdrawn and supernatant obtained by centrifugation at 13,000 rpm for 10 min. Samples of 1 μl of 1:3 supernatant dilutions were mixed with 200 μl Stains-all assay solution [14] and the absorbance was read at 438 nm using a PowerWave™ XS Microplate Spectrophotometer (BioTek). New calibration curves (0–0.1% w/v SDS) were acquired with each set of samples. In all the cases, samples were analysed by triplicate.

### MIC determination

The MIC for different compounds was determined by the dilution method in NB. A bacterial inoculum was prepared by diluting 100 times in NB a 0.5 McFarland suspension (10^8^ CFU/ml) from an exponential-phase culture (OD_600_: 0.5). One ml of the cell suspension was added to each tube containing the appropriate dilution of the tested compound. Then, cultures were incubated for 24 h at 28°C and 250 rpm. Growth inhibition was evaluated macroscopically, except for SDS, which was determined by serial dilutions and plate counts. The MIC value corresponds to the first dilution of each compound where no growth at all was observed. MIC values were tested in triplicate.

### Animal experiments

Animal experiments were performed in accordance with the European legislation governing animal welfare, and they were authorized and supervised by the Ethics Committee of Universidad de Oviedo. Rainbow trout (*Oncorhynchus mykiss*) of about 10–15 g obtained from a commercial fish farm were used in all the experiments. Fish were kept in 60 l tanks at 18°C ± 1 in dechlorinated water. Each batch was microbiologically analysed for potential pathogens before and during all the experimental process.

For LD_50_ determination, *Y. ruckeri* parental and *yraS* mutant strains cultures were grown to an OD_600_ of 0.5-0.6 (OD_600_ 0.5 = 10^8^ CFU/ml), harvested by centrifugation and washed twice with PBS. Cells were re-suspended in PBS and logarithmic dilutions were prepared. Groups of 10 fish were challenged by intraperitoneal injection of 100 μl of each dilution from 10^2^ to 10^8^ CFU/ml. Mortality was monitored daily over a 7-days period, and LD_50_ was calculated according to the PROBIT method using the SPSS statistical package for Windows, establishing a 95% confidence limit. LD_50_ experiments were performed in duplicate. Aliquots of bacterial suspensions injected in each dilution were plated on NA and after incubation for two days at 18°C colonies were counted for further LD_50_ determination. Dead animals were examined bacteriologically to confirm the presence of *Y. ruckeri*. Control fishes were injected with an equal volume of PBS.

### *In silico* analysis

Sequences were compared to those in the databases with the BLAST (Basic Local Alignment Search Tool) program. Protein sequences were aligned with MUSCLE http://www.ebi.ac.uk/Tools/msa/muscle/ [36] using default parameters. Alignment was manually inspected to correct inaccurately situated residues and a maximum likelihood tree was built with MEGA software http://www.megasoftware.net/ [37], using WAG + G model [38] as most suitable amino acid substitution method and 4 Gamma categories. Bootstrap analysis was performed by resampling 500 times. Topology was checked and edited using iTOL http://itol.embl.de/ [39,40] and manual graphical corrections were performed using Adobe Illustrator CS6 (Adobe Systems, USA).

### Availability of supporting data

The *yraS* sequence was deposited in GenBank under accession number KF421132.

## Additional files

Additional file 1: Figure S1 GC-MS spectra of the components found in the culture supernatant after incubation of *Y. ruckeri. Y. ruckeri* parental and yraS mutant strains were grown for 24 h at 28°C in NB containing 0.25% SDS. After 24 h of incubation at 28°C, cultures were filtrated. Filtrates were dried by vacuum centrifuge and finally resuspended in chloroform. Samples of 1μl of 1:400 dilutions were then analysed by GC-MS; framing the peak amplified in graph E. **(A)** Commercial 1-dodecanol (control). **(B)** Sample from *Y. ruckeri* 150 culture. **(C)** Sample from *Y. ruckeri* yraS mutant culture. **(D)** Sample from culture medium without inoculation. **(E)** Magnification of the peak relative to the retention time corresponding to 1-dodecanol from the sample of the *Y. ruckeri* parental strain culture. Additional file 2: Figure S2 Phylogenetic tree based on the deduced amino acid sequence of the YraS protein from *Y. ruckeri*. The protein alignment was carried out by the MUSCLE program [14], and further corrected manually and a phylogenetic tree of maximum likelihood was constructed using the MEGA program [36]. The topology was edited with the iTOL program [38,39]. Additional file 3: Figure S3 SDS-PAGE of cell extracts of different *Y. ruckeri* strains after Sudan black staining. Electrophoresis was performed at 15 mA in a cool room for 16 h. Then, the gel was incubated at 20°C for 4 h and stained with Sudan black dye. Cell extract from: lane 1, *Y. ruckeri* parental strain; lane 2, yraS^-^; lane 3, yraS^+^. Molecular masses are indicated in kDa on the left side of the gel. Only strains bearing the *yraS* gene were positive for the presence of the 120 kDa band stained with Sudan black. Additional file 4: Figure S4 Mean cumulative percent mortality of rainbow trout following challenge with *Y. ruckeri* parental and *yraS* mutant strains. Single groups of 10 fish were challenged by intraperitoneal infection with 1.8 × 10^2^ (■), 3.5 × 10^2^ (), 1.8 × 10^4^ (▲) and 3.5 × 10^4^ () CFU of parental (continuous line) and yraS mutant (dotted line) strains, and mortality was monitored every day. Additional file 5: Figure S5 Effect of glucose on the HSF^+^ phenotype of *Y. ruckeri*. Plates containing M9C with 0.5% w/v SDS **(A)**; and the same medium plus 1% w/v of glucose **(B)** were spotted with 5 μl of early stationary phase cultures of *Y. ruckeri* parental and yraS^-^ strains. After 96 h of incubation at 28°C, plates were photographed. The HSF^+^ phenotype, indicated by the creamy white colony corresponding to the parental strain grown in the absence of glucose **(A)**, was converted to HSF^-^ in the presence of glucose **(B)**.
