# Supplementary figures and images for: The heat sensitive factor (HSF) of Yersinia ruckeri is produced by an alkyl sulphatase involved in sodium dodecyl sulphate (SDS) degradation but not in virulence
Source: BMC Microbiol. 2014 Sep 30;14:221. doi: 10.1186/s12866-014-0221-7 (PMC4207315; doi:10.1186/s12866-014-0221-7)

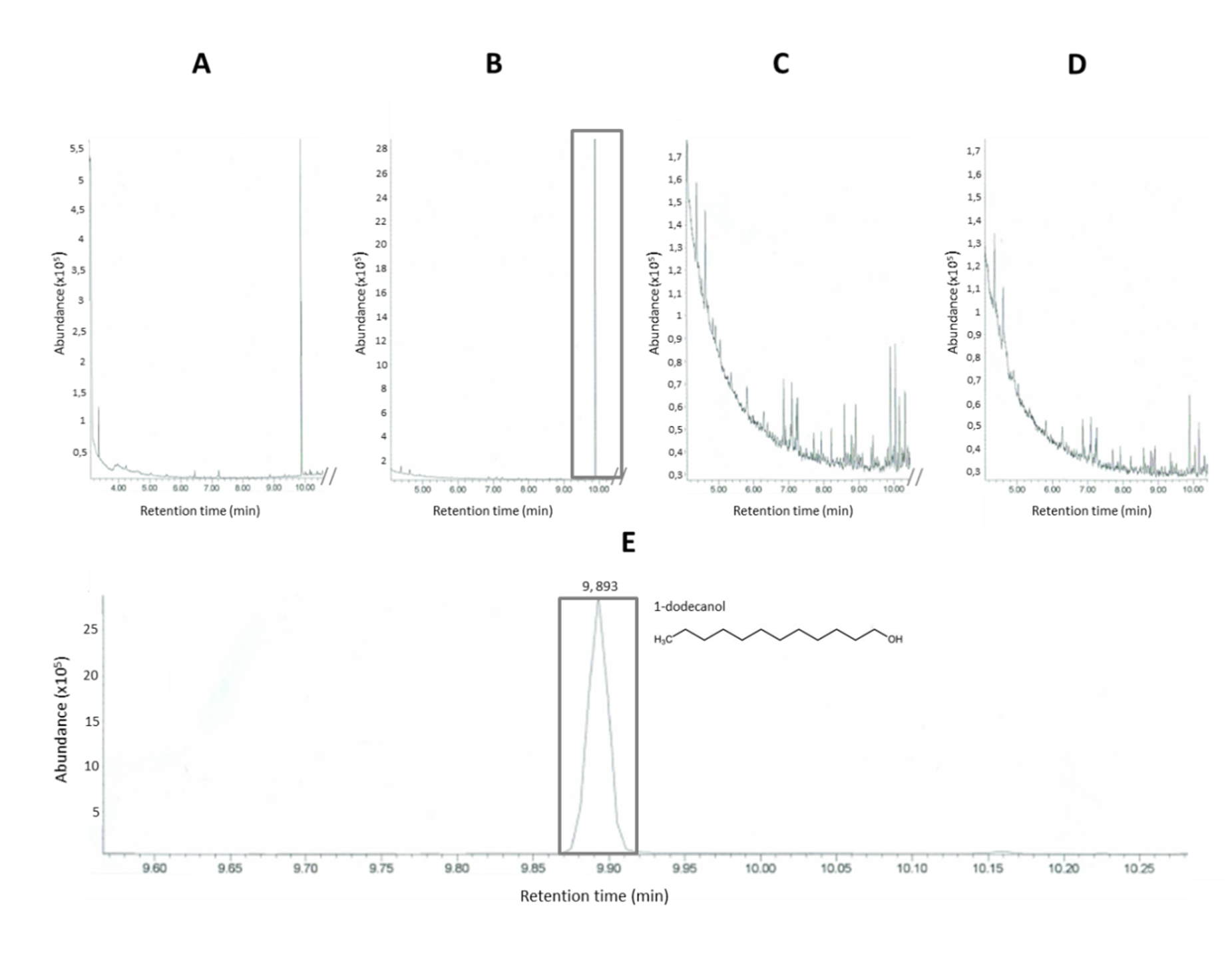

Supplement: Additional file 1: Figure S1 — GC-MS spectra of the components found in the culture supernatant after incubation of Y. ruckeri. Y. ruckeri parental and yraS mutant strains were grown for 24 h at 28°C in NB containing 0.25% SDS. After 24 h of incubation at 28°C, cultures were filtrated. Filtrates were dried by vacuum centrifuge and finally resuspended in chloroform. Samples of 1μl of 1:400 dilutions were then analysed by GC-MS; framing the peak amplified in graph E. (A) Commercial 1-dodecanol (control). (B) Sample from Y. ruckeri 150 culture. (C) Sample from Y. ruckeri yraS mutant culture. (D) Sample from culture medium without inoculation. (E) Magnification of the peak relative to the retention time corresponding to 1-dodecanol from the sample of the Y. ruckeri parental strain culture. [file 12866_2014_221_MOESM1_ESM.tiff]

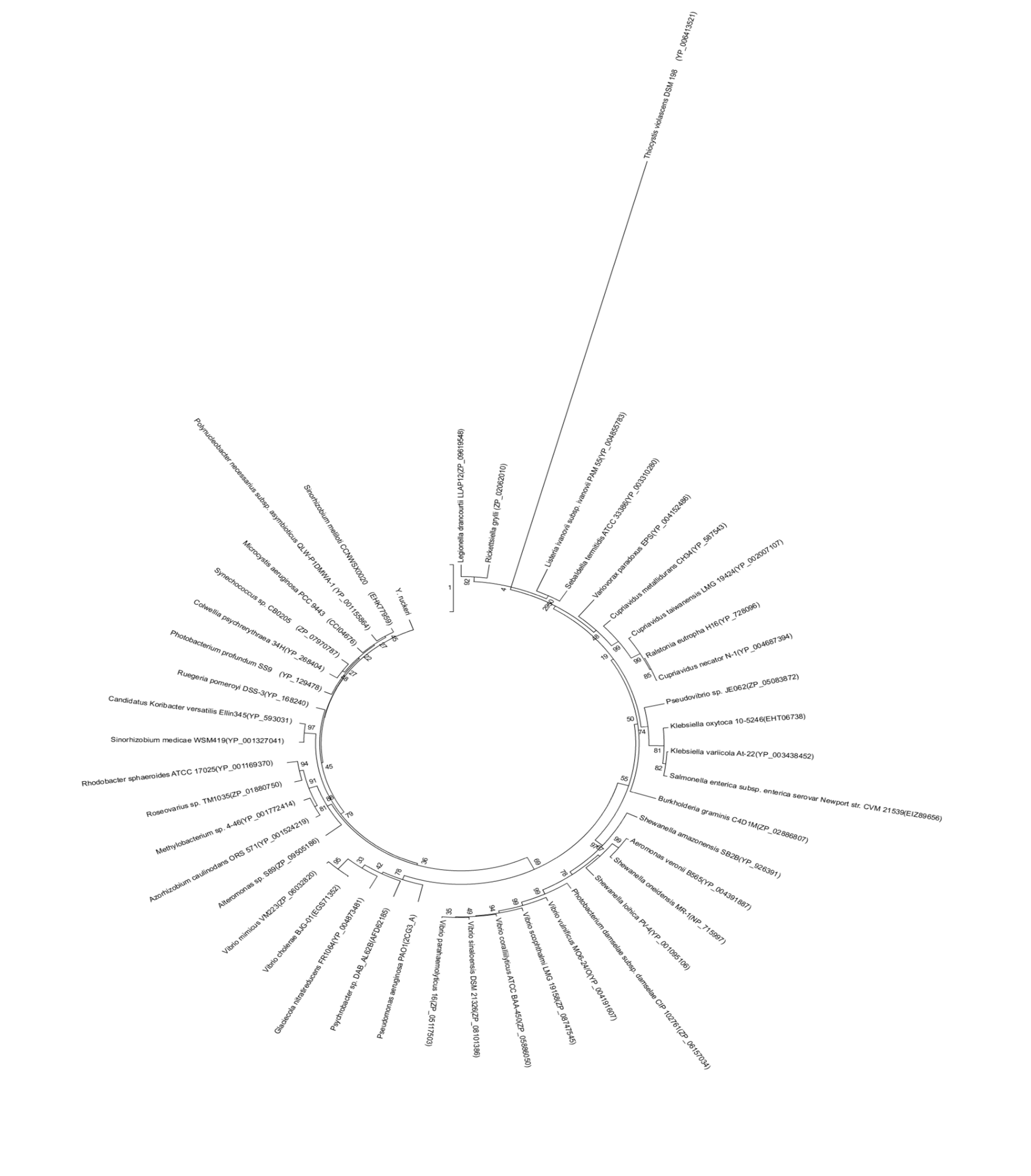

Supplement: Additional file 2: Figure S2 — Phylogenetic tree based on the deduced amino acid sequence of the YraS protein from Y. ruckeri. The protein alignment was carried out by the MUSCLE program [14], and further corrected manually and a phylogenetic tree of maximum likelihood was constructed using the MEGA program [36]. The topology was edited with the iTOL program [38,39]. [file 12866_2014_221_MOESM2_ESM.tiff]

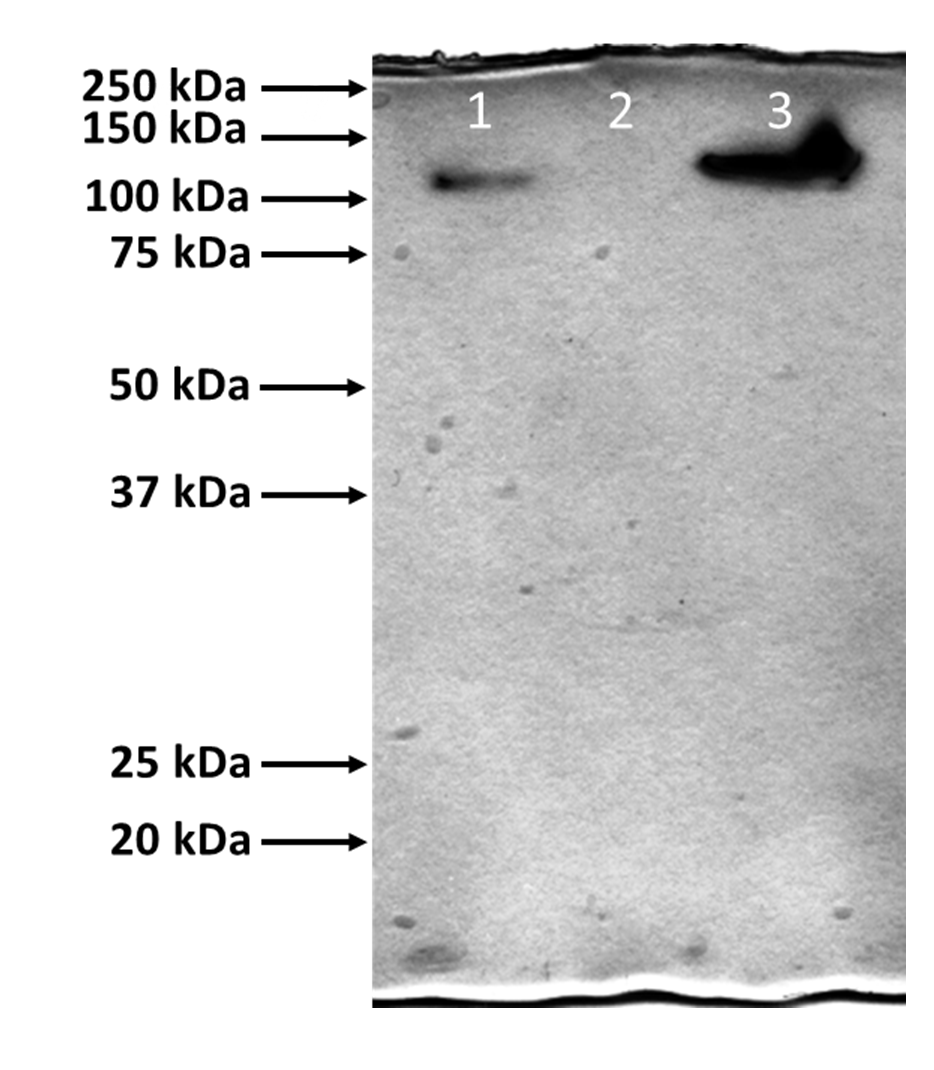

Supplement: Additional file 3: Figure S3 — SDS-PAGE of cell extracts of different Y. ruckeri strains after Sudan black staining. Electrophoresis was performed at 15 mA in a cool room for 16 h. Then, the gel was incubated at 20°C for 4 h and stained with Sudan black dye. Cell extract from: lane 1, Y. ruckeri parental strain; lane 2, yraS-; lane 3, yraS+. Molecular masses are indicated in kDa on the left side of the gel. Only strains bearing the yraS gene were positive for the presence of the 120 kDa band stained with Sudan black. [file 12866_2014_221_MOESM3_ESM.tiff]

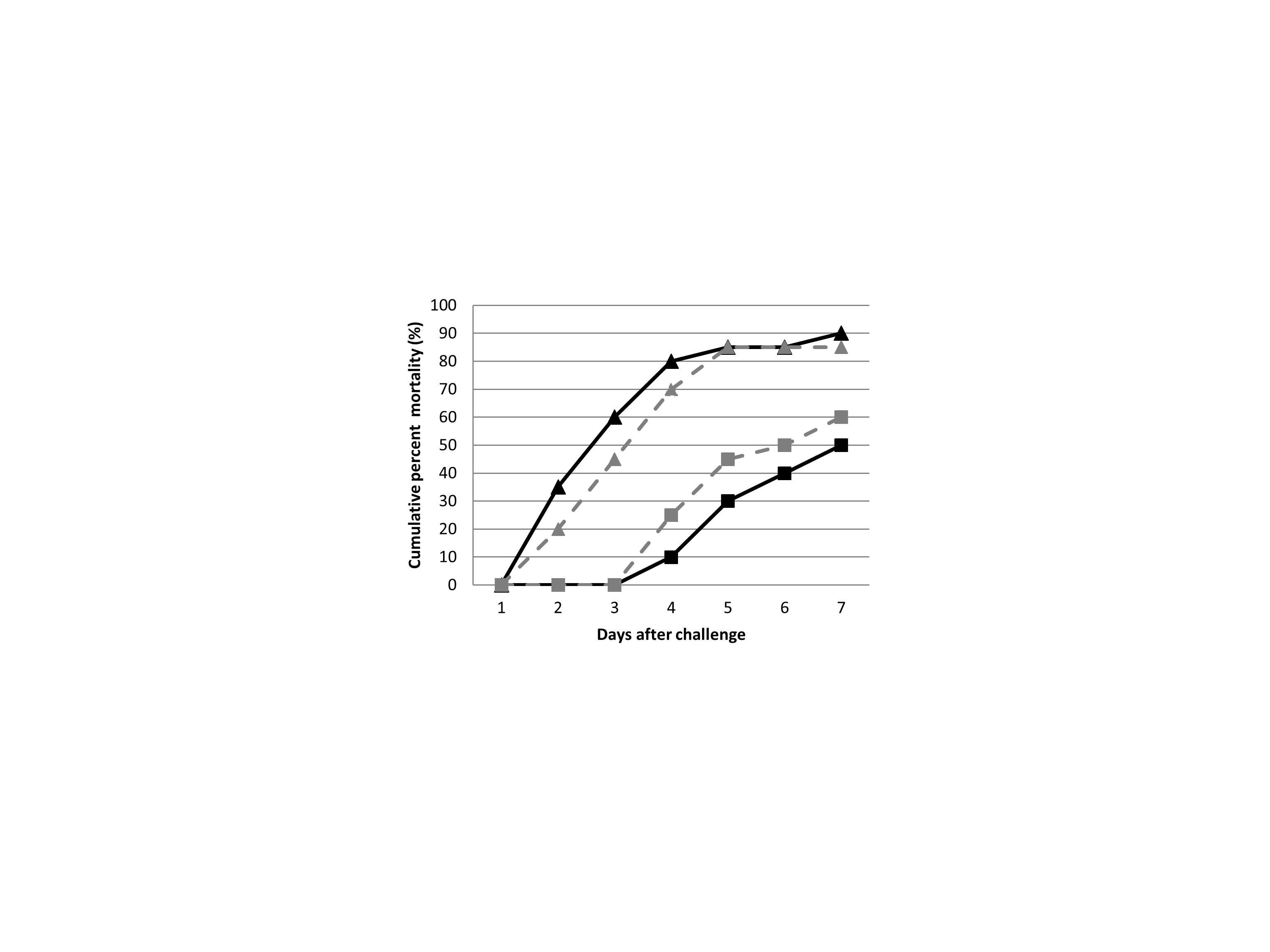

Supplement: Additional file 4: Figure S4 — Mean cumulative percent mortality of rainbow trout following challenge with Y. ruckeri parental and yraS mutant strains. Single groups of 10 fish were challenged by intraperitoneal infection with 1.8 × 10^2^ (■), 3.5 × 10^2^ (), 1.8 × 10^4^ (▲) and 3.5 × 10^4^ () CFU of parental (continuous line) and yraS mutant (dotted line) strains, and mortality was monitored every day. [file 12866_2014_221_MOESM4_ESM.tiff]

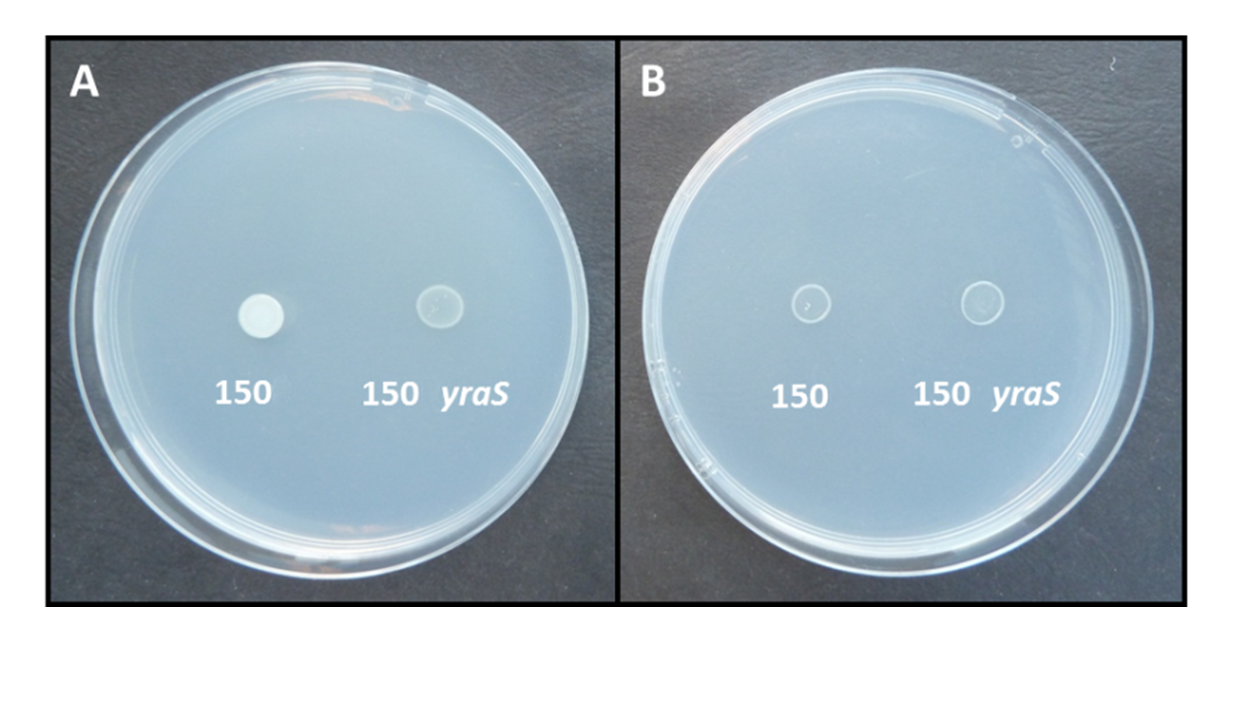

Supplement: Additional file 5: Figure S5 — Effect of glucose on the HSF+ phenotype of Y. ruckeri. Plates containing M9C with 0.5% w/v SDS (A); and the same medium plus 1% w/v of glucose (B) were spotted with 5 μl of early stationary phase cultures of Y. ruckeri parental and yraS- strains. After 96 h of incubation at 28°C, plates were photographed. The HSF+ phenotype, indicated by the creamy white colony corresponding to the parental strain grown in the absence of glucose (A), was converted to HSF- in the presence of glucose (B). [file 12866_2014_221_MOESM5_ESM.tiff]
